# Proteomic Definitions of Mesenchymal Stem Cells

**DOI:** 10.4061/2011/704256

**Published:** 2011-03-03

**Authors:** Martin H. Maurer

**Affiliations:** Department of Physiology and Pathophysiology, University of Heidelberg, Im Neuenheimer Feld 326, 69120 Heidelberg, Germany

## Abstract

Mesenchymal stem cells (MSCs) are pluripotent cells isolated from the bone marrow and various other organs. They are able to proliferate and self-renew, as well as to give rise to progeny of at least the osteogenic, chondrogenic, and adipogenic lineages. Despite this functional definition, MSCs can also be defined by their expression of a distinct set of cell surface markers. In the current paper, studies investigating the proteome of human MSCs are reviewed with the aim to identify common protein markers of MSCs. The proteomic analysis of MSCs revealed a distinct set of proteins representing the basic molecular inventory, including proteins for (i) cell surface markers, (ii) the responsiveness to growth factors, (iii) the reuse of developmental signaling cascades in adult stem cells, (iv) the interaction with molecules of the extracellular matrix, (v) the expression of genes regulating transcription and translation, (vi) the control of the cell number, and (vii) the protection against cellular stress.

## 1. Why Do We Study MSC Proteomes?

**Figure 1:**
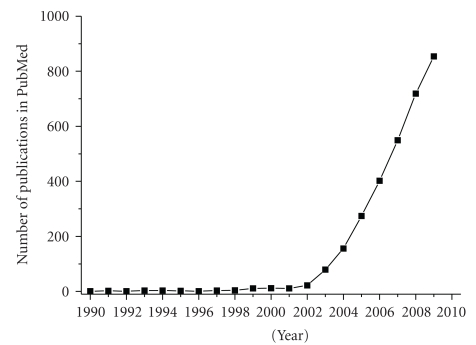
Scientific publications in PubMed about MSCs. The PubMed database was searched for the terms “mesenchymal stem cell,” “mesenchymal stromal cell,” and “MSC” for the years 1990–2009. The emerging interest in MSCs is seen by the increasing number of publications.

The human bone marrow consists of a heterogenous group of cells, including hematopoietic stem cells (HSCs), endothelial cells, fibroblasts, adipocytes, and osteogenic cells. Additionally, in recent years, another group of cells has been found which are able to proliferate and self-renew, as well as differentiate into cells of the mesenchym such as osteoblasts, chondrocytes, and adipocytes (Figure 1). These cells were called mesenchymal stem cells (MSCs), but there is a significant controversy about a consensus definition of MSCs—which at least can be seen in the large number of names for these cells, all used simultaneously, for example, mesenchymal stem cells, mesenchymal stromal cells, marrow stromal cells, mesodermal progenitor cells, marrow-isolated adult multilineage inducible (MIAMI) cells, or CFU-F (colony-forming unit fibroblastic) (reviewed and discussed in [1]). 

The interest in MSCs has risen since there is hope to use these cells in regenerative medicine, for example, in acute myocardial infarction and cardiovascular disease, diabetes, stroke, kidney disease, or immunomodulatory disease such as graft-versus-host disease (GvHD) [2–4]. Therefore, it is necessary to know the set of proteins which is responsible for the cellular functions. In recent years, proteomics emerged as a large-scale screening tool for both creating a protein inventory and to identify protein functionality. Major techniques of proteomics include two-dimensional gel electrophoresis for the separation of proteins and various mass spectrometry techniques for sequence identification [5]. 

In this paper, I will concentrate on the proteomic analysis of human MSCs, explicitly excluding MSCs isolated from other species (with several exceptions), or other stem cells differentiated into mesenchymal tissues in a strict sense, which are bone, cartilage, and fat, as well as MSCs differentiated into nonmesenchymal tissues such as neural or glial cells.

## 2. The Dilemma of a Consensus Definition of Mesenchymal Stem Cells: Heterogenous Origins and Properties

A functional definition of MSCs seems to be a straightforward approach, with MSCs fulfilling the following two criteria: (i) MSCs can be propagated in cell culture, where they adhere to the plastic well surface, and (ii) they can be stimulated to differentiate into osteoblasts, chondrocytes, and adipocytes. This definition is similar to the functional definition of other stem cell types, for example, for neural stem cells [6]. Of note, this definition excludes the transdifferentiation potential of MSCs into stem cells of other lineages, such as hematopoietic stem cells (HSCs), neural stem cells (NSCs), or epithelial stem cells. The concept of stem cell transdifferentiation is still under discussion and may be an artificial one [1, 7–10].

Besides the bone marrow, cells with regenerative potential have been isolated from the skeletal muscle [15], adipose tissue [16], umbilical cord [17], dental pulp [18], synovia [19], the circulatory system [20], and amniotic fluid [21] as well as fetal blood, liver, bone marrow, and lung [22–24]. The list (exceeding more than 30 organs by now) only shows that the tissue of origin cannot be used for the characterization of MSCs. Thus, we will concentrate in this paper on cells isolated from the bone marrow (BM-MSCs).

## 3. Cytochemical and Immunochemical Characterization

The cellular morphology played a major part in identifying MSCs *in vitro*. In general, immature MSCs appear as small, spindle-shaped cells, whereas mature MSCs display as larger cells with a flat, polygonal morphology. With regard to cellular staining techniques, MSCs were stained positive with sudan black, alkaline phosphatase, collagen IV, and fibronectin, whereas they are negative to esterase staining [25, 26]. 

**Figure 2:**
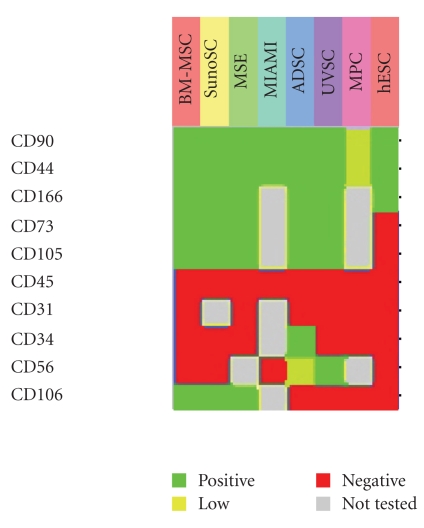
Expression of cell surface markers of MSCs. Hierarchical clustering of the expression of cell surface markers of MSCs from different MSCs preparations. Cells were termed “mesenchymal stem cells (MSCs)" [11], “mesodermal progenitor cells (MPCs)" [12], “marrow-isolated adult multilineage inducible (MIAMI) cells" [13], and “bone marrow-isolated mesenchymal stem cells (BM-MSCs)," “adipose tissue-isolated stem cells (ADSCs)," “synovia-derived stem cells (SynoSCs)," “umbilical vein stem cells (UVSCs)," and “human embryonic stem cells (hESCs)" [14]. The figure shows the different cell preparations in columns and the surface antigens in rows. The green color codes for positive expression, yellow codes for low expression, red codes for no expression, and grey color stands for not determined in the respective experiments. It is seen that the cells can be defined by the presence and absence of a distinct pattern of cell surface markers, that is, CD34(−), CD45(−), CD117(−) (cKit), CD44(+), CD90(+), and CD166(+).

With regard to immunocytochemical methods, the murine IgM monoclonal antibody STRO-1 identified bone marrow stromal cells distinct from HSCs [27]. Soon, several other surface proteins were identified which are specific for BM-MSCs [28]. In recent years, a panel of cell surface antigens emerged to characterize MSCs (compiled in [4]; see also Figure 2). According to these panels, negative and positive selection markers of MSCs have been defined. MSCs do not express CD34, CD45, CD117 (cKit), HLA class I, and HLA-DR antigens, whereas they are positive for CD13, CD29, CD44, CD73, CD90, CD105, and CD166. These markers are located in the cell membrane of the MSCs, and antibodies are readily available for FACS analysis and sorting.

## 4. Proteomics of Bone Marrow-Derived MSCs

### 4.1. Proteome Inventories

In a recent review, the basic protein inventory of a “typical” MSC has been compiled [29], including specific proteins of the cellular metabolism, ion transport, and their respective receptors, or channels, cytokines, chemokines, growth factors, and their respective receptors, proteins of stroma-dependent hematopoiesis, extracellular matrix and connective tissue proteins, calcium homeostasis, cell cycle regulation and cellular aging, transport proteins, protein for posttranslational protein modifications, cellular detoxification, genomic transcription and translation pathways, a specific set of cell surface molecules (see also Figure 2), differentiation and development, structural components and cytoskeleton, and folding of proteins and stress response proteins (chaperones). Although no single molecule has been identified as a specific marker for MSCs, and all proteins of the list have also been found in other cell expression studies, the set of proteins and their expression arrangement is specific for MSCs.

Comparing two different approaches for MSC culture conditions, Wagner et al. [30] isolated MSCs from the bone marrow of healthy human donors and performed 2DE and subsequent MS to identify a set of 136 proteins. The authors compared the proteomic data to gene chip profiling data for an integrated approach and found, after interchanging media between the two groups after 8 days, that culture conditions reversed the gene and protein expression profiles. In conclusion, it is important to control and report culture conditions in details, since these experimental conditions highly influence gene and protein expression. This makes it difficult to compare experiments with different cell culture conditions [31].

A recent study compared the cellular proteomes of MSCs isolated from bone marrow (BM-MSC), adipose tissue (ADSC), synovial membrane (SynoSC), and umbilical vein wall (UVSC) [14], finding a close correlation of protein expression patterns between BM-MSCs and ADSCs. The authors used cells with the immunophenotype CD90(+), CD73(+), CD105(+), CD44(+), CD45(+) for BM-MSC, ADSC, SynoSC, and UVSC. Additionally, ADSC expressed CD34, and UVSC did not express CD106. The authors used 2DE to separate about 850 protein spots, of which 232 could be identified by MS. 

The proteomic comparison of rat BM-MSCs from young and old animals [32] revealed the differential expression of Beta-actin FE-3, Caldesmon l, Calponin-l, E-FABP (C-FABP), Galectin 3, Gamma synuclein, Heterogeneous nuclear ribonucleoprotein Al isoform a and A2/Bl isoform A2, Huntingtin interacting protein K, Myosin light chain, Peroxiredoxin 5, Pyruvate dehydrogenase (lipoamide) beta, and Transgelin. These proteins can be divided into the functional groups of cytoskeletal rearrangement, cellular ageing, and metabolism. Moreover, these proteins were also expressed under osteogenic differentiation. 

A problem of MSC cultures is that the cells cannot be propagated over a longer period of time because of intrinsic differentiation programs. Within a few passages, the MSCs lose their proliferation potential. In a proteomic study using 2DE and LC-MS/MS, Lee at al. [33] found that the addition of 10 ng/mL basic fibroblast growth factor (bFGF) differentially regulated the expression of 15 proteins, of which actin-related protein 2/3 complex subunit 2 (ARPC2), isoform 2 of glial fibrillary acidic protein (GFAP), lamin-A/C (LMNA), ubiquinol-cytochrome c reductase complex core protein 1 (UQCRC1), the multifunctional protein ADE2 (PAICS), F-actin-capping protein subunits alpha-1 (CAPZA1) and alpha-2 (CAPZA1), Septin-2 (SEPT2), and elongation factor 1-gamma (EEF1G) have been upregulated and Myosin regulatory light chain (MRLC2), desmoplakin (DSP), proteasome subunit alpha type 5 (PSMA5), and heat shock protein beta-1 (HSPB1) have been downregulated more than 2-fold. The functional classification of these proteins showed that these proteins mainly belonged to structural and cell morphology regulating groups, indicating that bFGF might regulate MSC differentiation and structure.

### 4.2. Myogenic Differentiation

Wang et al. used commercially available MSCs which are positive for CD105, CD166, CD29, and CD44, and negative for CD34, CD14, and CD45 in MSCGM media complemented with fetal bovine serum [34]. They cultured the MSCs up to ten passages before 2DE. For myogenic differentiation, the authors stimulated the cells for four days with 10 ng/mL TGF-*β* and identified about 30 proteins with differential expression, among them smooth muscle actin-alpha and gelsolin. 

To induce myogenic differentiation in human MSCs, Kurpinski et al. [35] used 5 ng/mL TGF-*β*1 and uniaxial mechanical stress. After 24 h, the authors subjected the cells to 2DE and Q-TOF (quadrupole time of flight) MS. They identified 12 proteins which were upregulated, including eukaryotic translation elongation factor 2 (EF2), transforming growth factor-beta-induced protein ig-h3 (BGH3), calponin 3 (CNN3), tropomyosin, actin-related protein 3 (ARP3), and smooth muscle actin-gamma. All of these proteins may be involved in myogenesis.

In a proteomic study investigating the secretome of murine BM-MSCs by LC-MS/MS, Sarojini et al. [36] identified 19 proteins, including fibronectin, pigment epithelium-derived factor (PEDF), collagen A2(I), myocilin, clusterin, protein-lysine 6-oxidase, biglycan, cathepsin L, peptidyl-prolyl cis-transisomerase, nucleobindin, procollagen c-proteinase enhancer protein, collagen A1(I) chain, Dickkopf-related protein-3 (Dkk-3), fibulin-2, *β*-2-microglobulin, CTLA-2-alpha protein, cystatin c, galectin-3, and moesin. In a cell migration assay, the secretome was applied to human fibroblast cultures. In this assay, the chemotactic migration towards to PEDF-containing secretome was increased, suggesting a function in tissue rearrangement.

### 4.3. Adipogenic Differentiation

The adipogenic differentiation of commercially available human MSCs was investigated by the addition of 10% FBS, 1 *μ*M dexamethasone, 0.5 mM methyl-isobutylxanthine, 10 *μ*g/mL h-insulin, and 10 mM indomethacin to the cell culture media for 14 days [37]. The MSCs expressed CD90, but not CD34 and CD45. After 2DE and MALDI-TOF/MS, the authors identified syntaxin binding protein, oxysterol binding protein-3- (OSBP-) related protein, phosphodiesterase PDE9A12, glycophorin, immunoglobulin kappa chain variable region, PPAR-gamma, and T-cell receptor V-beta 4 as new or overexpressed protein spots. 

In commercially available BM-MSCs, which are CD14(−), CD29(+), CD34(−), CD44(+), CD45(−), CD105(+), and CD166(+), Ju et al. investigated the whole cell proteome and membrane proteome by 2D-DIGE and LC-MS/MS during adipogenic differentiation [38]. Cells were incubated for 3 weeks in the LG-DMEM differentiation media containing 0.5 mM 3-isobutyl-1-methylxantine, 1 mM hydrocortisone, 0.1 mM indomethacin, and 10% FBS. The authors identified more than 700 proteins, including 33 CD marker proteins, most of which are already known for MSCs, a large number of solute carriers, and several integrins, which are specialized cell surface molecules for cell-cell interaction and adhesion to the extracellular matrix. Moreover, more than 30 proteins were only identified in the differentiated MSCs, most of which were metabolic enzymes.

### 4.4. Osteogenic Differentiation

In a one-dimensional proteomic study based on polyacrylamide gel electrophoresis (SDS-PAGE) protein separation and LC-MS/MS, Chiellini et al. [39] searched “secretome", that is, the cell culture supernatant, for secreted proteins of human multipotent adipose tissue-derived stem (hMADS) cells during adipogenesis and osteogenesis. The authors listed 73 proteins in the secretome, of which the plasminogen activator inhibitor PAI-1 was found only in the osteogenic differentiation group, implicating a possible regulator between adipogenesis and osteogenesis. 

Interestingly, the transplantation of MSCs into the transgenic PAI-1^−/−^ mouse increased graft survival [40]. The authors also analyzed the MSC cell culture supernatant after hypoxia by LC-MS/MS, finding 11 upregulated proteins, including brevican, IGF binding proteins 2, 4, and 5, myeloperoxidase, ceruloplasmin, biglycan, serine proteinase inhibitor El (PAI-1), vascular endothelial growth factor A, apelin, and superoxide dismutase 3, and 9 downregulated proteins, including IGF-2 binding protein 3, serine hydroxymethyl transferase 1, hepatoma-derived growth factor, chemokine (CC motif) ligand 2, desmoplakin, apolipoprotein D, Kit oncogene, secreted frizzled-related sequence protein 1, and interferon zeta. 

Sun et al. [41] subcultured BM-MSCs for 14 days in osteogenic media containing 10 mM *β*-glycerophosphate, 100 nM dexamethasone, and 50 mg/mL ascorbic acid-2-phosphate. They compared protein extracts by 2DE and MS to undifferentiated BM-MSCs, mapping more than 1,000 proteins on the gels. They found the upregulation of 8 proteins, including Annexin A1 and A2, Pyruvate kinase 3 (muscle), Enolase 1, Heat shock 27 kD protein 1, Protein disulfide isomerase-related protein, Proteasome 26S ATPase subunit 5, and Cathepsin D, as well as the downregulation of 4 proteins, including T-complex protein 1, Proteasome 26S ATPase subunit 2, Cadherin-2, and Chaperonin containing TCP1, subunit 3. Moreover, the authors compared serial subcultures to construct a time-resolved expression pattern for the MSC subcultures.

Using an immortalized human telomerase reverse transcriptase transduced MSC line (MSC-TERT), Foster et al. enriched protein extracts for membrane proteins and searched for differentially expressed proteins after osteoblastic differentiation by liquid chromatography (LC) and MS [42]. The authors identified 463 proteins, including MSCs phenotype CD71, CD105, CD166, CD44, Thy1, CD29, and CD63. Upon differentiation, the expression of 83 proteins increased, including alkaline phosphatase (ALP), versican core protein, and tenascin, and 21 proteins decreased more than 2-fold, including fatty acid synthase. 

Kratchmarova et al. [43] investigated the effects of 50 ng/mL EGF, or 10 ng/mL PDGF for 3 days on the osteogenic differentiation of hMSC-TERT cells by LC-MS/MS. Whereas EGF stimulated bone formation, PDGF did not. Proteins which were found only in the EGF-treated cells included EGFR, ErbB2, c-Cbl, DOC-2, Acid phosphatase 1, Ribonuclease inhibitor, CYLD, and KIAA2002, whereas proteins only found in the PDGF-treated group included PDGFR alpha and beta, PI-3K (p85-alpha, p85-beta, p110-alpha, p110-beta, p110-delta), Fyn, and Protocadherin 43.

### 4.5. MSCs and Cancer Proteomics

Whereas the self-renewal and multipotency of stem cells largely contribute to the hope of cell and tissue regeneration strategies in disease, recent data nourished the hypothesis that stem cells may also contribute to tumor generation. Their cancer-generating potential is not only seen in embryonic stem cells (ESCs), but also in MSCs, or in cells with similar potential, termed cancer-associated fibroblasts (CAFs) and circulating endothelial progenitors (CEPs) [44–46]. Therefore, the protein analysis of tumor-generating stem cells may contribute to the mechanisms of tumorigenesis.

With regard to the proteomic analysis of leukemic stromal cells, BM-MSCs from healthy donors were compared to leukemic cells using liquid phase IEF, 2D-DIGE, MALDI-MS/MS, and iTRAQ methods [48, 49]. The author found more than 900 proteins in the MSC samples by liquid phase IEF and resolved more than 5,000 protein spots on the 2D gels. In total, 34 proteins showed increased expression in leukemic stromal cells compared to nonleukemic MSCs, whereas 39 proteins showed decreased expression. Most of the differentially expressed proteins belonged to proteins with functions in transcription and metabolic regulation.

## 5. Proteomics of Umbilical Cord-Derived MSCs (UCB-MSCs)

MSCs can also be isolated from the umbilical cord blood by the same means as BM-MSCs [50]. Whereas the functional criteria remain comparable (adhesion to the plastic surface), cell surface markers differ largely and can be used to distinguish between BM-MSCs and UCB-MSCs.

**Figure 3:**
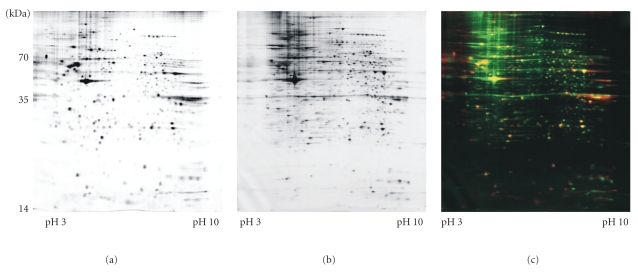
Comparison of 2DE gels of BM-MSCs and UCB-MSCs. This figure shows representative 2D gel images of (a) bone marrow (BM-)MSC proteins and (b) umbilical cord blood (UCB-)MSC proteins. (c) The false-colored overlay image of the two 2D gels shows the BM-MSC proteins colored in red, and the UCB-MSC proteins colored in green. The resulting overlaid color is yellow (data from [30, 47]). The overlaid gel images show a close relationship between the two cellular proteomes, but there seem to be more proteins in the BM-MSC group as well as some differences in protein expression, as seen in the unique color in one of the gels.

One of the first studies creating a proteomic inventory of umbilical cord-derived mesenchymal stem cells (UCB-MSCs) was from our own group [47]. We used CD29(+), CD44(+), CD73(+), CD90(+), HLA-class I(+), CD14(−), CD34(−), CD133(−), and HLA-class II(−). We separated more than 2045 protein spots by 2DE and identified 205 proteins by MS. The identified proteins could be grouped into functional categories, such as metabolism, folding, cytoskeleton, transcription, signal transduction, protein degradation, detoxification, vesicle/protein transport, cell cycle regulation, apoptosis, and calcium homeostasis. In the direct comparison, the proteomes of this UCB-MSC preparation and of BM-MSCs are largely congruent, but there are still distinct differences in the protein expression pattern (Figure 3). Thus, the concept of the protein definition of MSCs evolved accordingly to the NSC proteomic definition [6, 51].

A major problem of 2DE is that hydrophobic proteins of the cell surface cannot easily be isolated in the same sample preparation as the hydrophilic proteins [5]. With regard to MSCs, Jeong et al. described the proteome preparation of hydrophobic proteins from UCB-MSCs [52]. The authors identified 35 proteins, providing additional insight into the cell surface composition of UCB-MSCs. 

A recent study compared the proteomes of UCB-MSCs, BM-MSCs, and placenta-derived MSCs by 2DE and MS [53]. The immunophenotype of all cell preparations was CD29(+), CD44(+), CD90(+), CD105(+), CD166(+), CD45(−), HLA-DR(−), CD3(−), CD16(−), CD19(−), CD33(−), CD38(−), CD34(−), and CD133(−). All cell preparations could be differentiated into bone and fat cells. The authors identified six differentially regulated proteins, that is, superoxide dismutase [Mn] (MnSOD), heat shock protein HSPA9, cathepsins B and D, prohibitin, and plasminogen activator inhibitor PAI-1. 

Roche et al. did not use MSCs from the umbilical cord blood, but from the umbilical cord vein walls [14]. The authors compared the proteomes to other MSC preparations (see discussion above).

## 6. The Concept of Dedifferentiation Is Still a Matter of Debate

There is one proteomic study based on 2DE and MS investigating the dedifferentiation potential of murine fibroblasts, which showed similar expression patterns with regard to cytoskeletal and cell shape remodeling, RNA export, degradation, folding, stress control, and ATP production [54]. Although dedifferentiation was initiated using the synthetic purine “reversine" (2-(4-morpholinoanilino)-6-cyclohexylaminopurine), the general concept of dedifferentiation is still under discussion [55–57], also in the respective proteomic study, since no redifferentiation experiments showing the proposed multipotentiality of the dedifferentiated cells have been shown.

## 7. Molecular Definitions of MSCs Using 2DE Experiments

With regard to proteomic analyses, proteomic inventories of MSCs have been created using two-dimensional gel electrophoresis. Additionally, the events underlying cellular differentiation *in vitro* have been studied in osteogenesis, adipogenesis, and chondrogenesis. 

**Figure 4:**
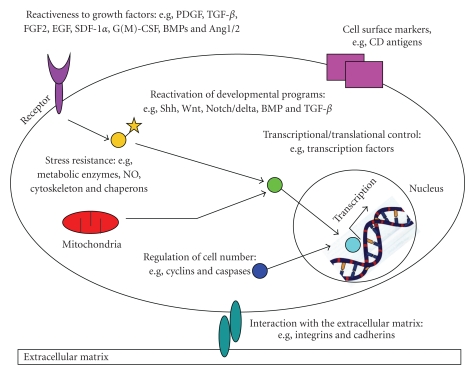
Proteomic definition of MSCs—proposal for the protein inventory. The main aspects for a proteomic definition of MSCs include (i) the expression of a specific cell surface markers, (ii) the reactiveness to growth factors, (iii) the re-activation of developmental pathways, (iv) the interaction with the extracellular matrix, (v) the regulation of transcription and translation, (vi) the regulation of cell number, and (vii) the machinery for resistance to cellular stress (modified from [6, 51]).

Comparing the protein expression patterns, no single characteristic molecule could be identified, but the functional definition, as specified for the genomic approaches, is supported. In the light of these results, adult stem cells have all the same prerequisites, but the exact molecular composition is dependent on cell and tissue-specific factors [51, 58]. These common prerequisites involve (Figure 4) 

(i) The expression of a specific set of cell surface markers, such as CD13, CD29, CD44, CD73, CD90, CD105, and CD166, and the absence of hematopoietic stem cell markers, such as CD34, CD45, CD117 (cKit), HLA class I, and HLA-DR antigens.

(ii) The responsiveness to growth factors and cytokines such as PDGF, TGF-*β*, FGF [59], EGF, SDF-1alpha, G-CSF, GM-CSF, Angiopoietin-1, Angiopoietin-2, BMP-4, BMP-7, and IFN-gamma (reviewed in [60–62]). Of note, not all mesenchymal stem cells react to each of these molecules. A prerequisite to growth factor responsiveness is the expression of specific membrane-spanning receptors, which are able to initiate intracellular signaling cascades.

(iii) The reuse of developmental signaling cascades in adult progenitors. Nearly, 20 such pathways have been identified in adult stem cells, including Shh, Wnt, Notch/Delta, BMP, and TGF-*β* (reviewed in [60, 61, 63]). Interestingly, the activation of these pathways in the adult may have different functions compared to the embryonic developmental pathways.

(iv) The interaction with molecules of the extracellular matrix (reviewed in [64, 65]). During differentiation and maturation, stem cells migrate, extend processes, attach to the extracellular matrix, and adhere to nearby microenvironmental surfaces. Therefore, they require the expression of motor proteins, lytic enzymes, and enzymes for providing metabolic energy. Several extracellular matrix proteins mediating cell-cell interaction have been identified in mesenchymal stem cells, such as integrins and cadherins.

(v) The expression of genes regulating transcription and translation (reviewed in [66–69]). Although no specific proteins in the categories of transcription factors, DNA or RNA binding proteins, and chromatin remodeling enzymes can be named, mesenchymal stem cells need these molecules to change the cellular phenotype from its undifferentiated form to the new functional requirements of a differentiated mature cell.

(vi) Mechanisms for controlling cell number. Mainly three processes regulate stem numbers: Mitosis (proliferation), differentiation, and apoptosis (programmed cell death). An essential process is also asymmetric cell division, which regulates the stem cell pool. In this context, typical proteins regulating cell number are caspases and cyclins. 

(vii) The protection against cellular stress (reviewed in [70]), which involves metabolic deprivation, NO and O_2_ toxicity, DNA damage, mechanical distortion, hypo- and hyperthermia, or hypoxia. Proteins in this category have been identified in the groups of molecular chaperones, NO-detoxifying enzymes, and components of the proteasome. 

Of note, although mesenchymal stem cells express these molecules, different proteins can be found in different cellular phenotypes. This heterogeneity in the protein expression pattern is supported by protein expression studies comparing mesenchymal stem cells isolated from different organs [14].

## 8. Conclusions

Human MSCs represent a heterogenous group of stem cells with a unique potential to differentiate into mesenchymal tissue. In this paper, proteomic experiments investigating human MSCs have been reviewed with the purpose to find common protein expression patterns. Six functional groups of proteins have been identified which build the prerequisite of an MSC proteomics inventory, including proteins for growth factor responsiveness, reuse of developmental pathways, regulation of transcription and translation, mechanisms controlling cell numbers, and protection against cellular stress. The composition of this set is unique, but not exclusive, for human MSCs.

## Abbreviations

2DE: Two-dimensional gel electrophoresis
ADSC: Adipose tissue-isolated stem cells
BM-MSC: Bone marrow-derived mesenchymal stem/stromal cell
DIGE: Differential gel electrophoresis
FACS: Fluorescence-activated cell sorting
hESC: Human embryonic stem cells
hMADS: Human multipotent adipose tissue-derived stem cells
HSC: Hematopoietic stem cell
iTRAQ: Isobaric tags for relative and absolute quantification
LC: Liquid chromatography
MS: Mass spectrometry
MS/MS: Tandem mass spectrometry
MSC: Mesenchymal stem/stromal cell
NSC: Neural stem cell
Q-TOF: Quadrupole time-of-flight
SDS-PAGE: Sodium dodecyl sulfate polyacrylamide gel electrophoresis
SynoSC: Synovia-derived stem cells
UCB: Umbilical cord blood
UVSC: Umbilical vein stem cells
CAF: Cancer-associated fibroblasts
CEP: Circulating endothelial progenitors.
